# The Role of Cholesterol in the Interaction of the Lipid Monolayer with the Endocrine Disruptor Bisphenol-A

**DOI:** 10.3390/membranes12080729

**Published:** 2022-07-23

**Authors:** Victoria M. Katata, Mateus D. Maximino, Carla Y. Silva, Priscila Alessio

**Affiliations:** Department of Physics, School of Technology and Applied Sciences, São Paulo State University (UNESP), Presidente Prudente 19060-080, SP, Brazil; victoria.katata@unesp.br (V.M.K.); mateus.maximino@unesp.br (M.D.M.); carla.yasmim@unesp.br (C.Y.S.)

**Keywords:** bisphenol-A, Langmuir films, membrane models, endocrine disruptors

## Abstract

Among pollutants of emerging concern, endocrine disruptors (ED) have been shown to cause side effects in humans and animals. Bisphenol-A (BPA) is an ED by-product of the plastic industry and one of the chemicals with the highest volume produced yearly. Here, we studied the role of cholesterol in the BPA exposure effects over membrane models. We used Langmuir films of both neat lipid DPPC (1,2-dipalmitoyl-sn-glycero-3-phosphocholine) and cholesterol (Chol) and a binary mixture containing DPPC/Chol, exposing it to BPA. We evaluate changes in the π-A isotherms and the PM–IRRAS (polarization modulation–infrared reflection adsorption spectroscopy) spectra. BPA exposure induced changes in the DPPC and Chol neat monolayers, causing mean molecular area expansion and altering profiles. However, at high surface pressure, the BPA was expelled from the air–water interface. For the DPPC/Chol mixture, BPA caused expansion throughout the whole compression, indicating that BPA is present at the monolayer interface. The PM–IRRAS analysis showed that BPA interacted with the phosphate group of DPPC through hydrogen bonding, which caused the area’s expansion. Such evidence might be biologically relevant to better understand the mechanism of action of BPA in cell membranes once phosphatidylcholines and Chol are found in mammalian membranes.

## 1. Introduction

In the last few years, concern with the water quality has been growing due to its contamination with substances, which in many cases have unknown side effects. These substances are called emerging pollutants, which, in many cases, are not entirely removed from the drinking water even after the treatment, causing all sorts of issues to human health and the environment [1]. Among those substances, there are drugs, plasticizers, flame retardants, hygiene products, and many other organic compounds generated by human activities [2,3]. Some of those compounds have the capacity to cause harmful effects on the endocrine system of humans and animals, even at low concentrations. Such a group of substances is known as endocrine disruptors (ED), and such compounds can mimic or emulate the natural action of hormones in the organism [4]. The groups highly vulnerable to exposure to EDs are children and pregnant women, due to their developmental stages of life that require a balanced production of hormones. Once the endocrine system is affected, many health issues are reported, such as obesity, diabetes, thyroid dysfunction, cancers, and other diseases [5].

A well-known endocrine disruptor is bisphenol-A (BPA). BPA is used to fabricate plastics and epoxy resins and is highly produced, reaching three million tons per year worldwide [1]. Studies demonstrate that BPA leaches into food and beverages, therefore being ingested frequently [6]. Many studies report the harmful effects caused by BPA contamination, such as prostate and breast cancer, early puberty, and infertility [7,8,9]. As much as studies report the side effects of BPA on human and animal health, there are still gaps in the molecular mechanism of action in the cell. The literature studies report the capacity that BPA must interact with the receptors of estrogens and androgens of the thyroid, for example [8]. Nevertheless, such systems are very complex, making it difficult to conclude how exactly BPA affects those structures [10,11].

The specificity of membrane models is why mimetic systems are ideal for identifying specific interactions at the molecular level between the lipid and external analytes. Using membrane models is the first step toward understanding how BPA interacts with the cells, since the membrane is the first contact point with external compounds [12]. The plasma membrane is composed of glycerophospholipids and sterols [13]. Cholesterol is a component of great importance in lipid membrane models, being responsible, for instance, for regulating water flow, thickness, and compressibility [14]. The dysregulation of normal cholesterol levels interrupts membrane functions [15]. Furthermore, the fluidity of the lipid layer is controlled by the presence of cholesterol, affecting the propagation or attenuation of signals that may occur across the membrane [16].

Here, we investigate the role of cholesterol in the interaction of mimetic membranes composed of DPPC, a phospholipid of the phosphatidylcholines class, and BPA. The choice of mimetic membrane based on DPPC and cholesterol was based on comparing how BPA affects a liquid-ordered phase model (DPPC/Chol mixture) compared to a gel-phase model (neat DPPC), since BPA had been demonstrated to affect fluid models more significantly.

## 2. Materials and Methods

### 2.1. Reagents

**Figure 1:**
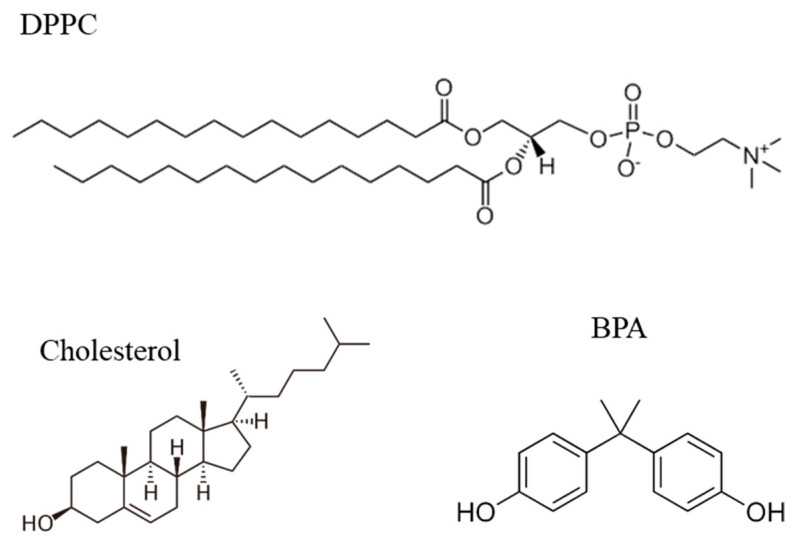
Chemical structures of DPPC, cholesterol, and BPA.

For this experiment, 1,2-dipalmitoyl-sn-glycero-3-phosphocholine (DPPC) with 99% purity and cholesterol were purchased from Avanti Polar Lipids, Inc. (Alabaster, AL, USA). Bisphenol-A (BPA, purity ≥ 90%) was purchased from Sigma Chemical Co. (St. Louis, MO, USA), while chloroform was obtained from Merck (Darmstadt, Germany). The lipid solutions (DPPC and Chol) were prepared in chloroform, both at the same concentration of 1 mM. The lipid mixture was produced by adding three parts of DPPC and one part from cholesterol, achieving a 3:1 ratio. BPA aqueous solutions were obtained with ultrapure Milli-Q water (resistivity 18.2 MΩ·cm, surface tension 72 mN/m at 25 °C), and pH = 5.6. The ultrapure water was used in all membrane models as a standard to observe only the interactions between DPPC and BPA. The chemical structures of DPPC, Chol, and BPA are shown in Figure 1.

### 2.2. Langmuir Films

Langmuir films of DPPC, cholesterol, or DPPC/Chol mixtures (3:1) (1 mM in chloroform) were spread onto ultrapure water (Milli-Q) and in the presence of 1 × 10^−5^ mol/L BPA subphase with a KSV Nima (Espoo, Finland) trough model 2000, whose volume was 220 mL. The films were characterized using surface pressure vs. mean molecular arear (π-A) isotherms, taken 15 min after spreading to allow chloroform evaporation. The π-A isotherms were measured using a Wilhelmy sensor (Espoo, Finland) to measure the surface tension while compressing the monolayer with two symmetric barriers at a rate of 10 mm/min. All experiments were performed in triplicate at 23 ± 1 °C (Figure S1—Supplementary Materials).

### 2.3. PM–IRRAS

Polarization–modulated infrared reflection absorption spectroscopy (PM–IRRAS) analysis was carried out using a KSV PMI 550 spectrometer (KSV Instruments Ltd., Helsinki, Finland). This spectrometer contains a HgCdTe detector (MCT, model PCI-3TE-10.6), which works with an active area of 1 mm^2^. The incident infrared beam was modulated with a ZnSe photoelastic modulator at a resonance frequency of 50 kHz. A background measurement was taken to reduce the effect of the water in the spectra. The experiment was similar to the π-A isotherms, but with the PM–IRRAS spectra only taken at the surface pressure of 30 mN/m. The BPA concentration used was 10^−5^ mol/L. The incident beam angle modulated between parallel (p-polarized) and perpendicular (s-polarized) polarizations for all the measurements was 80° relative to the normal. The experiments were performed in the spectral range from 800 to 4000 cm^−1^ and with a spectral resolution of 8 cm^−1^.

## 3. Results and Discussion

### 3.1. Langmuir Monolayers

**Figure 2:**
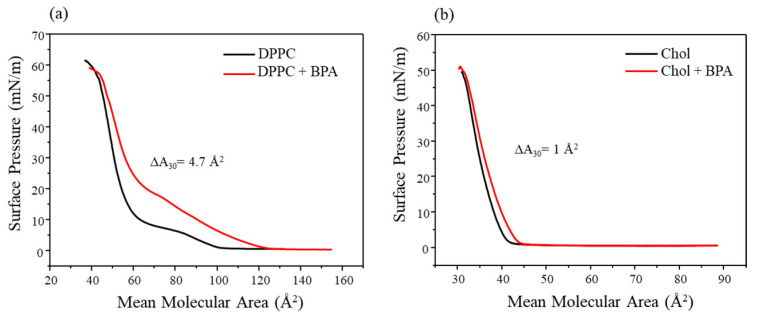
π-A isotherm for (**a**) DPPC and (**b**) cholesterol Langmuir films in ultrapure water subphase and the presence of BPA (1 × 10^−5^ mol/L).

The π-A isotherms of DPPC in ultrapure water (Figure 2a) presented the expected profile for the lipid, as reported in the literature [17], with the clear phase transitions from liquid-expanded to liquid-condensed phase. In the presence of 1 × 10^−5^ mol/L of BPA, the DPPC monolayer presented a displacement to bigger areas during almost all of the compression, indicating that BPA remained at the air–water interface among the lipid molecules. Furthermore, the analyte altered the profile of the isotherm if compared to the neat DPPC in water. Such behavior was expected from BPA due to its surface activity, which can be attributed to its bolaamphiphilic properties, which consist of the extremities of the molecules having hydrophilic behavior and the center having hydrophobic behavior [18], allowing the molecule to behave like a surfactant while being soluble in water. BPA insertion at the subphase promoted a variation in the extrapolated area of 13.8% (from 59.56 ± 0.63 to 67.8 ± 0.11 Å^2^). However, once the high surface pressures were achieved and the monolayer packing increased, the BPA molecules were partially expelled from the air–water interface, promoting a rearrangement in the lipid molecules (Figure 2a).

This result agrees with the literature. For instance, Maximino et al. [19] studied a similar system, exposing DPPC monolayers to BPA. The authors observed that BPA induced an expansion in the monolayer while changing the isotherm profile. Such behavior of the lipid monolayer after the insertion of an analyte in the subphase was also observed by Ruiz et al. [20], where the influence of the hormone 17 α-ethinylestradiol (another endocrine system disruptor) in binary membrane models using DPPC and cholesterol was studied. In the analysis containing 100% DPPC and exposed to the hormone, the result was similar to that presented in this work, with the same pattern of displacement, consisting of the expansion at lower pressures and consequent expulsion from the interface at higher pressures.

π-A isotherms of cholesterol in ultrapure water (Figure 2b) showed a more compact monolayer, evidenced by the abrupt increase in surface pressure when the mean molecular area decreased, which is a behavior present in rigid monolayers, as seen in the literature [20]. The cholesterol molecules start packing at about 40 Å^2^, being rigid and not obtaining the liquid-expanded phase present in the isotherms of pure DPPC. In the presence of BPA, there is a small displacement to larger areas, starting the packing in the range of 45 Å^2^. This effect can be attributed to the small cholesterol molecule having high rigidity and very compact packaging, not allowing the BPA molecules to remain at the air–water interface. This is evidenced by the variation in the area (ΔA) at 30 mN/m, which was only 1 Å^2^, demonstrating that BPA promotes little effect on the cholesterol monolayer. A similar result was obtained by Wyzga et al. [21], where the influence of BPA and its derivatives on monolayers of some lipids and cholesterol was studied. The effect of the presence of BPA was similar to that obtained in this work, with BPA showing a slight expansion of the monolayer and unchanging the isotherm profile in the presence of BPA, as shown in Figure 2b. The study of neat Chol is in the manuscript mainly to compare to the other samples and investigate specific interactions/effects between the membrane models and BPA.

**Figure 3:**
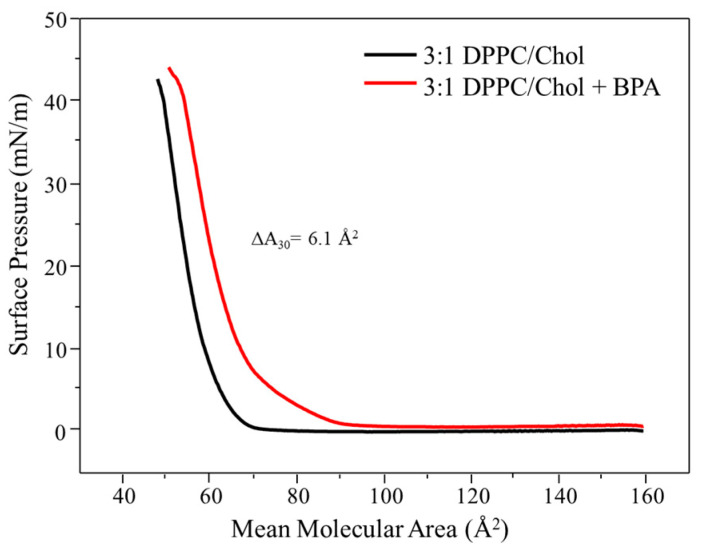
π-A isotherm for the mixture of DPPC and cholesterol (3:1) Langmuir film in ultrapure water subphase and the presence of BPA (1 × 10^−5^ mol/L).

Isotherms in ultrapure water of the DPPC/Chol mixture were studied in a 3:1 ratio, as shown in Figure 3.

DPPC was in greater quantity because it belongs to the phosphatidylcholine class, the major component in the plasma membrane of mammals [13]. The cholesterol in the mixture induces a condensation in the DPPC monolayers [20], enhancing its packing and practically making the characteristic plateau of DPPC isotherms disappear. The mean molecular area for the mixture to start packing is in the range of 70 Å^2^. With the exposure of BPA in the DPPC/cholesterol monolayer subphase, the isotherm shifted to higher mean molecular area values during the entire compression. The presence of BPA promoted an increase of 11.6% in the area (from 51.6 Å^2^ to 57.7 Å^2^ at 30 mN/m), indicating that BPA was not expelled from the monolayer interface even at the highest surface pressures, thus remaining between the lipid molecules. Despite the expansion of the monolayer, the isotherm profile was retained, suggesting that BPA did not affect the fluidity of the monolayer. Similar behavior was observed in work by Ruiz et al. [20], where the influence of the hormone 17 α-ethinylestradiol on mixed monolayers of DPPC and cholesterol in different proportions was studied. A similar result was observed for the proportion with the percentage closest to this work (30% cholesterol), where the isotherm shifted to larger areas in the presence of the analyte during the entire compression. Moreover, among all the studied proportions, the 30% sample presented the slightest variation in fluidity, similar to this manuscript.

Some interesting data related to the influence of BPA on the lipid mixture lie in the fact that the DPPC/cholesterol monolayer presented the greatest area variation (ΔA) at a pressure of 30 mN/m (biologically relevant value due to lateral pressure of cells) [22] if compared to the neat monolayers (DPPC or Chol). The mixture presented a ΔA value of 6.1 Å^2^; meanwhile, for DPPC and cholesterol, the ΔA values were, respectively, 4.7 and 1 Å^2^. This result indicates that cholesterol plays an important role in the BPA interaction with the mimetic membrane. Furthermore, it is noteworthy that such a proportion of the mixture is close to the real composition of plasma membranes [13,23].

### 3.2. PM–IRRAS

**Figure 4:**
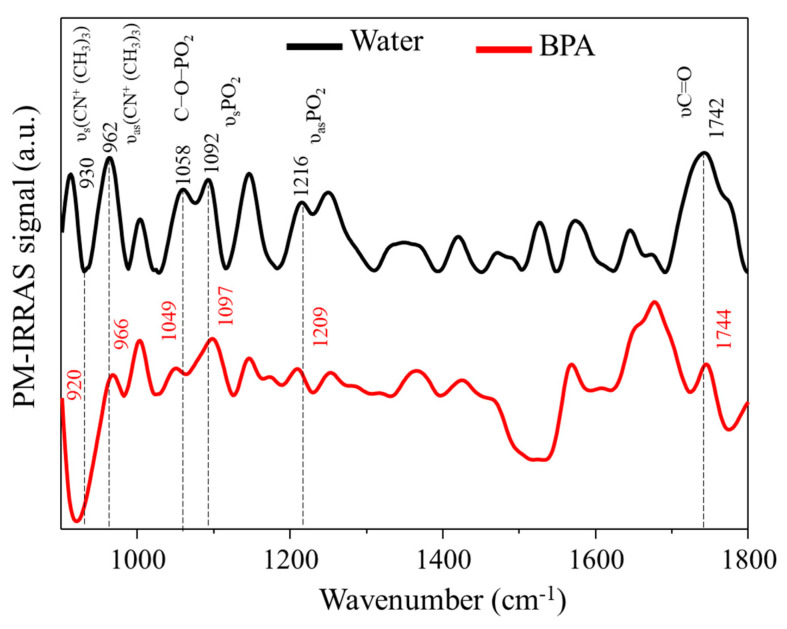
PM–IRRAS spectra from 900 to 1800 cm^−1^ for the DPPC/cholesterol mixture (3:1) in the absence and presence of BPA (1 × 10^−5^ mol/L) obtained at room temperature (23 °C) and surface pressure of 30 mN/m.

Figure 4 shows PM–IRRAS spectra for the DPPC/Chol (3:1) mixture in the absence and presence of BPA (1 × 10^−5^ mol/L).

The PM–IRRAS spectrum for the ultrapure water subphase (Figure 4—black line) showed the characteristic bands of DPPC traditionally reported in the literature [24,25]. Note the presence of the carbonyl group stretching (υ) (C=O, 1742 cm^−1^), in addition to the symmetrical (υ_s_) and antisymmetrical (υ_as_) bands of the phosphate group (P=O, υ_s_ = 1092 cm^−1^ and υ_as_ = 1216 cm^−1^), as well as the νCO-PO_2_ (1058 cm^−1^) bands and also the attributions of the symmetrical υs(CN+(CH_3_)_3_), 930 cm^−1^) and antisymmetric (υ_as_( CN^+^(CH_3_)_3_), 962 cm^−1^). In the presence of BPA, the bands of the υ_as_(CN^+^(CH_3_)_3_, υ_s_PO_2_, and υC=O groups did not undergo considerable displacement, indicating that the groups are the least affected in the presence of the pollutant. However, displacements were observed for the υC-O-PO_2_ and υ_as_PO_2_ groups, from 1058 to 1049 cm^−1^ and from 1216 to 1209 cm^−1^, respectively, indicating a possible interaction between the analyte and the monolayer. Such changes can be attributed to an interaction between BPA and the phospholipid head. The interaction is occurring through the phosphate group due to the observed changes; therefore, it is assumed that a hydrogen bond between the oxygen of the phosphate group of DPPC and the hydrogen of the phenol group of BPA is taking place. This type of interaction is widely discussed in the literature in studies of monolayers affected by pollutants. In the work of Maximino et al. [26], the influence of BPA on fluid monolayers of the DOPC lipid was studied. The authors obtained similar conclusions for the interaction between the BPA and lipid, mostly related to the phosphate group via hydrogen interactions. Furthermore, Hac-Wydro et al. [27] studied the effects of BPA and its derivatives on several monolayers, including POPC. The authors reached a similar conclusion, with BPA interacting with the lipid head via hydrogen interactions. A shift to the symmetrical choline band (from 930 to 920 cm^−1^) was also noted in their work. It is noteworthy that the changes observed in the choline groups may also be associated with a change in the conformation of the lipid head [28,29,30] due to changes induced by the interaction in the phosphate group [19,28].

**Figure 5:**
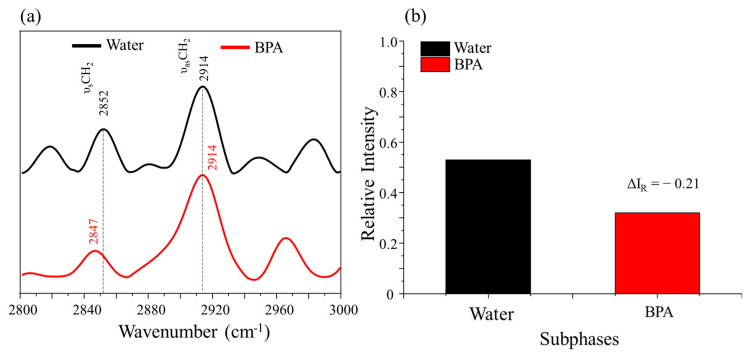
(**a**) PM–IRRAS spectrum from 2800 to 3000 cm^−1^ of the DPPC/cholesterol mixture (3:1) in the absence and presence of BPA (1 × 10^−5^ mol/L), obtained at room temperature (23 °C) and surface pressure of 30 mN/m. (**b**) Bar graph of the relative intensities ratio (ΔI_R_) of the symmetric and antisymmetric CH_2_ stretching calculated from PM–IRRAS spectra of DPPC/cholesterol mixture (3:1) in the absence and presence of BPA (1 × 10^−5^ mol/L).

PM–IRRAS spectra obtained for the DPPC/cholesterol mixture are shown in Figure 5a. In general, there was no significant variation in the vibration frequency for the bands related to υCH_2_ (symmetrical and antisymmetrical), suggesting no chemical interaction between BPA and the phospholipid tail. (Figure 5a). However, the order/disorder level in the lipid tail region can be assessed by analyzing the variation in the relative intensity between the CH_2_ bands (symmetrical and antisymmetrical). According to Levin et al. [31], the greater the relative intensity variation (ΔI_R_), the greater the degree of alteration in the order. If the ΔI_R_ values are positive, this indicates an increase in disorder; if the ΔI_R_ values are negative, this indicates an increase in order in the phospholipid tails at the air–water interface [32].

In the presence of BPA, there was a variation for negative values of ΔI_R_, which indicates that the presence of BPA is inducing a greater order in the tail region. Such a change may help explain the non-variation in the DPPC/cholesterol isotherms’ profile, since the ordering of the tails increased, it being understandable that the profile of the isotherms does not change. Therefore, this fact confirms that the expansion of the monolayers is linked to the interaction with the lipid head, the displacement matched by the presence of the analyte at the monolayer interface. The organization-increasing behavior induced by an external analyte was also observed by Ruiz et al. [20] in their study of DPPC/cholesterol monolayers exposed to the 17 α-ethinylestradiol, which showed the same negative variation in ΔI_R_ values. The authors concluded that the electrostatic interaction between EE2 and DPPC modified the rearrangement of the lipid chain in the interface.

**Figure 6:**
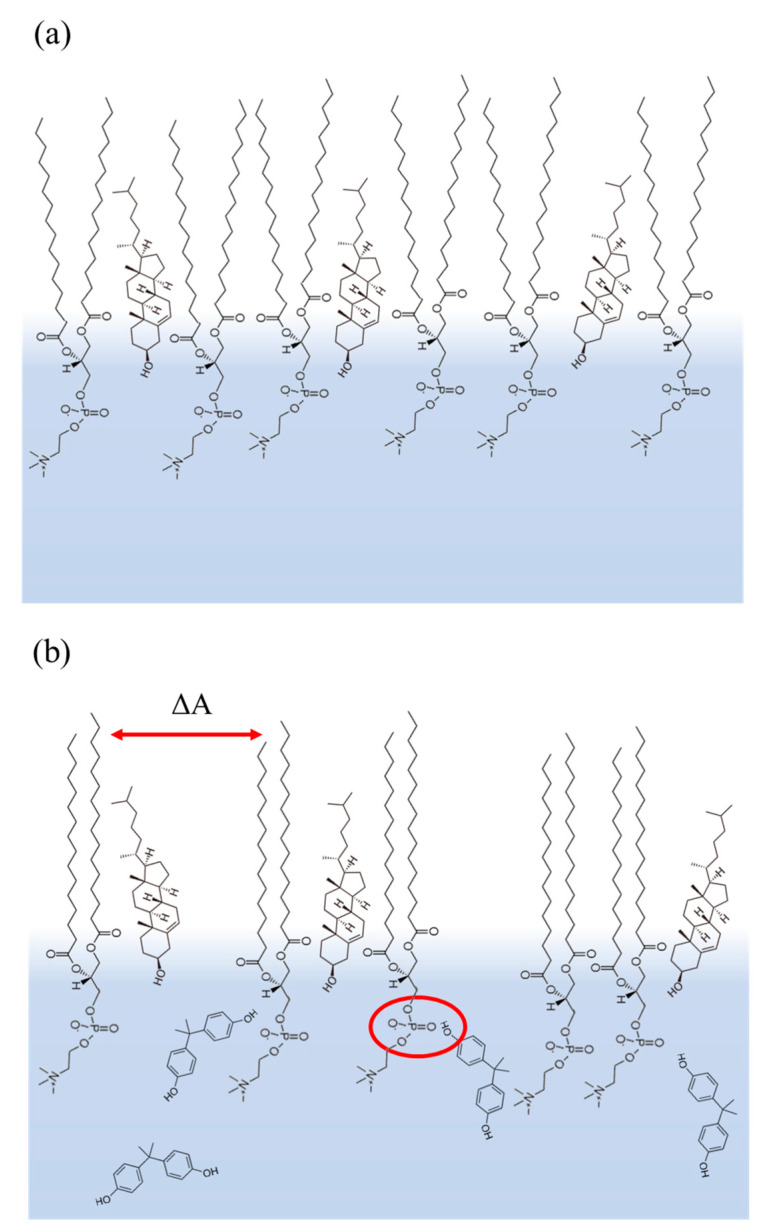
Monolayer structuring of DPPC/Chol (3:1) mixture at 30 mN/m in the (**a**) absence and (**b**) presence of BPA. The red circle indicates the possible interaction between the lipids and BPA.

Figure 6 summarizes all the effects observed in the π-A isotherms and the PM–IRRAS analysis into a proposed monolayer structuring for the lipids in the absence and presence of BPA.

Figure 6a displays the DPPC/Chol (3:1) mixture with the DPPC and cholesterol at the air-water interface, forming a packed and organized monolayer. As reported in some manuscripts, the cholesterol molecules remained near the lipid tails instead of the head group due to the ability to align its rings to the lipid hydrocarbon chain [33,34]. The tilt on the tail part of DPPC represents the higher disorder in the alkyl region observed in the PM–IRRAS analysis (Figure 5b). Once BPA was inserted in the subphase, its molecules occupied the area around the lipid head group (phosphate) due to the interactions described in the PM–IRRAS section, indicated by the red circle in Figure 6b, which caused the monolayer to expand up to 6.1 Å^2^ (ΔA), which was the highest variation at 30 mN/m, if compared to the neat lipids. The increased order at the alkyl chain was represented by the more upright position in the tails (Figure 6b). The presence of BPA at the monolayer is a combination of effects such as the interaction between BPA and the phosphate group of DPPC. The surface activity of BPA might have contributed to this effect, as well as the well-known lipophilicity of BPA, granting it a high affinity for lipids [35]. The role of cholesterol is probably related to the space between the lipids that facilitate the penetration and interaction of BPA since it restructures the DPPC monolayer, promoting greater organization.

## 4. Conclusions

In this paper, we studied the effects of BPA on mimetic membranes using binary monolayers composed of DPPC and cholesterol to reveal the role of cholesterol in the BPA–membrane interaction. We have found that at the lateral pressure of natural membranes (~30 mN/m), the presence of cholesterol induces the most significant displacement at the Langmuir isotherms when exposed to BPA. This behavior was associated with the monolayer’s organization, promoted by cholesterol. The organized monolayer results from DPPC and cholesterol interaction, as seen by PM–IRRAS, and makes more space available at the head region of the phospholipid, facilitating the BPA’s penetration.

## Data Availability

The data presented in this study are available on request from the corresponding author.

## Supplementary Materials

The following supporting information can be downloaded at: https://www.mdpi.com/article/10.3390/membranes12080729/s1, Figure S1: Triplicates of the π-A isotherms of (a,b) DPPC, (c,d) cholesterol, and (e,f) the mixture of DPPC/Chol in the absence and presence of BPA.
