# Supplementary material for: The Role of Cholesterol in the Interaction of the Lipid Monolayer with the Endocrine Disruptor Bisphenol-A
Source: Membranes (Basel). 2022 Jul 23;12(8):729. doi: 10.3390/membranes12080729 (PMC9332047; doi:10.3390/membranes12080729)
Supplement: Supplementary file 1 [file membranes-12-00729-s001.zip › membranes-1768207-supplementary.pdf]

membranes

MDPI

Supplementary Material

The Role of Cholesterol in the Interaction of the Lipid Monolayer with the Endocrine Disruptor Bisphenol-A

Victoria M. Katata, Mateus D. Maximino, Carla Y. Silva and Priscila Alessio *

Department of Physics, School of Technology and Applied Sciences, São Paulo State University (UNESP), Presidente Prudente 19060-080, SP, Brazil; victoria.katata@unesp.br (V.M.K.); mateus.maximino@unesp.br (M.D.M.); carla.yasmim@unesp.br (C.Y.S.)
* Correspondence: priscila.alessio@unesp.br

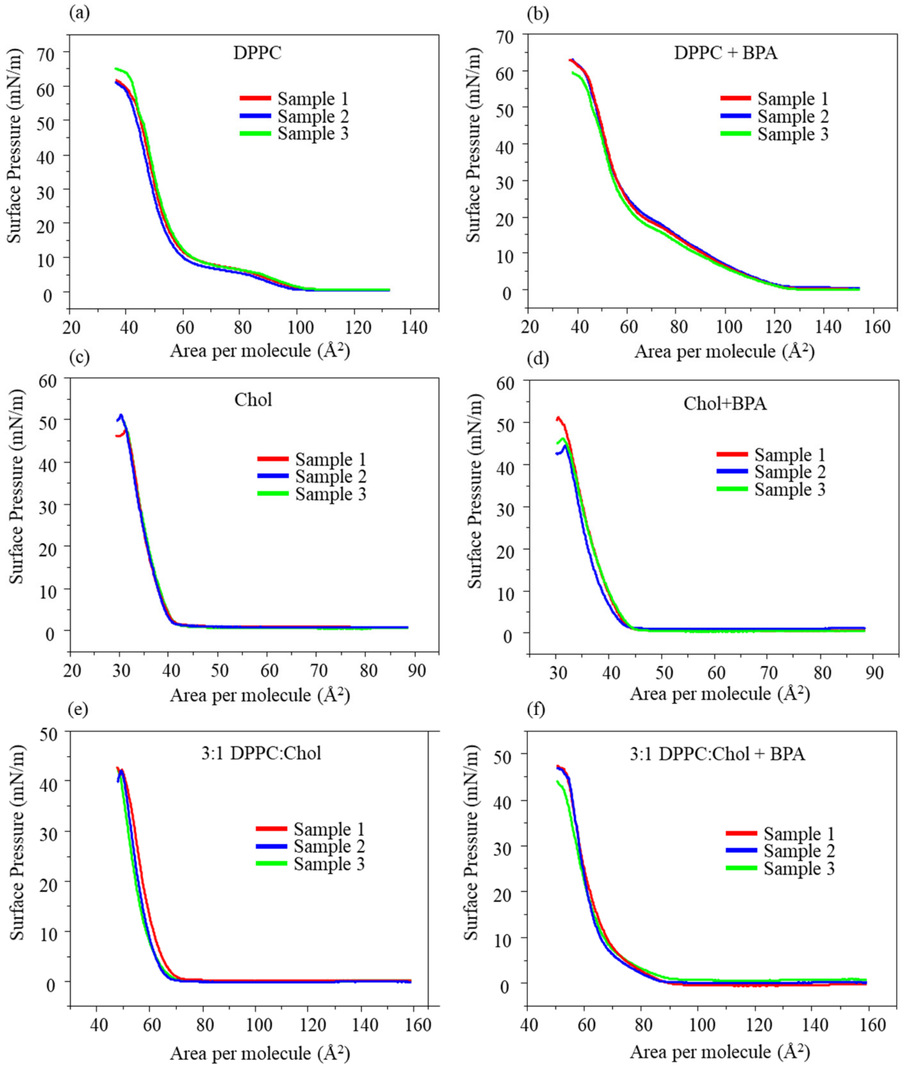

Figure S1. Triplicates of the π-A isotherms of (**a**,**b**) DPPC, (**c**,**d**) cholesterol, and (**e**,**f**) the mixture of DPPC/Chol in the absence and presence of BPA.

Citation: Katata, V.M.; Maximino, M.D.; Silva, C.Y.; Alessio, P. The Role of Cholesterol in the Interaction of the Lipid Monolayer with the Endocrine Disruptor Bisphenol-A. *Membranes* **2022**, *12*, 729. https://doi.org/10.3390/membranes12080729

Academic Editors: Lucia Sessa

Received: 27 May 2022
Accepted: 5 July 2022
Published: 23 July 2022

Publisher's Note: MDPI stays neutral with regard to jurisdictional claims in published maps and institutional affiliations.

Copyright: © 2022 by the authors. Submitted for possible open access publication under the terms and conditions of the Creative Commons Attribution (CC BY) license (https://creativecommons.org/licenses/by/4.0/).
